# De Novo Transcriptome Assembly in *Firmiana danxiaensis*, a Tree Species Endemic to the Danxia Landform

**DOI:** 10.1371/journal.pone.0139373

**Published:** 2015-10-01

**Authors:** Su-Fang Chen, Ming-Wan Li, Hui-Juan Jing, Ren-Chao Zhou, Gui-Li Yang, Wei Wu, Qiang Fan, Wen-Bo Liao

**Affiliations:** 1 State Key Laboratory of Biocontrol and Guangdong Provincial Key Laboratory of Plant Resources, School of Life Sciences, Sun Yat-sen University, Guangzhou 510275, China; 2 National Engineering Resarch Center of Plant Space Breeding, South China Agricultural University, Guangzhou 510642, China; 3 South China Botanical Garden, Chinese Academy of Science, Guangzhou 510650, China; The National Orchid Conservation Center of China; The Orchid Conservation & Research Center of Shenzhen, CHINA

## Abstract

Many *Firmiana* species are locally endemic, providing an interesting system for studying adaptation and speciation. Among these species, *F*. *danxiaensis* is a tree species endemic to Mount Danxia in Guangdong, China, which is an area known for presenting the Danxia landform. How *F*. *danxiaensis* could have adapted to the stressful environment of rocky cliffs covered with barren soils in the Danxia landform is still unknown. In this study, we performed de novo assembly of the transcriptome of *F*. *danxiaensis*, obtaining 47,221 unigenes with an N50 value of 987 bp. Homology analysis showed that 32,318 of the unigenes presented hits in the NCBI non-redundant database, and 31,857 exhibited significant matches with the protein database of *Theobroma cacao*. Gene Ontology (GO) annotation showed that hundreds of unigenes participated in responses to various stresses or nutritional starvation, which may help us to understand the adaptation of *F*. *danxiaensis* to Danxia landform. Additionally, we found 263 genes related to responses to Cd, partially explaining the high accumulation of Cd observed in *Firmiana* species. The EuKaryotic Orthologous Groups (KOG) and Kyoto Encyclopedia of Genes and Genomes (KEGG) annotations revealed many genes playing roles in the biosynthesis of secondary metabolites and environmental adaptation, which may also contribute to the survivor and success of *Firmiana* species in extreme environments. Based on the obtained transcriptome, we further identified a *Firmiana*-specific whole-genome duplication event that occurred approximately 20 Mya, which may have provided raw materials for the diversification of *Firmiana* species.

## Introduction

*Firmiana*, a small genus belonging to Malvaceae, contains only 12–18 species [1–4] (http://www.theplantlist.org/). Interestingly, all of the known *Firmiana* species except for *F*. *simplex* and *F*. *colorata* are locally endemic, such as *F*. *danxiaensis* occurring in Mount Danxia of Guangdong, China, *F*. *kwangsiensis* in the limestone mountains of Guangxi, China, *F*. *hainanensis* in the central and southern mountains of Hainan, China, and *F*. *minahassae* in Luzon Province, Philippines [1,4]. The molecular mechanisms underlying the speciation of these endemic *Firmiana* species have attracted scientific attention.

Mount Danxia is one of the famous examples of Danxia landform characterized by red-colored sandstones and conglomerates of largely Cretaceous age [5]. In the past three decades, five endemic plant species were found there, namely, *Firmiana danxiaensis*, *Lyonia danxiaensis*, *Chiritopsis danxiaensis*, *Danxiaorchis sinchiana*, and *Viola hybanthoides* [6–10]. The endemism in this unique Danxia landform also arouse people’s interests.

*F*. *danxiaensis*, mainly occurring in the thin soil of rocky stiff, is a dominant species of Mount Danxia. Tens of thousands of its individuals could be found there [6]. An investigation of the soil properties of Mount Danxia showed that its soils are highly acidic (pH = 4.47 ± 0.29) and barren, with low available nitrogen (54.43 ± 34.60 mg/kg), phosphorus (3.14 ± 5.41 mg/kg) and potassium (49.14 ± 34.68 mg/kg) [11]. Thus, the question arises of how *F*. *danxiaensis* has survived and become dominant in such a highly stressful environment. Furthermore, why is the distribution of *F*. *danxiaensis* restricted to Mount Danxia?

The current status of many *Firmiana* species could be very vulnerable, such as *F*. *major* and *F*. *hainanensis* were listed as threatened species in the IUCN Red Lists [12]. *F*. *danxiaensis* was listed as a threatened species in the China Species Red List [13], and also under second-class state protection in China [14]. Yet scientific researches about these species are quite limited due to the small quantity of publicly available sequence data. The rapid development of high-throughput sequencing technologies has paved the way for large-scale sequencing of non-model species in a cost-effective way [15–18]. In this study, we sequenced the transcriptome of *F*. *danxiaensis* using Illumina paired-end sequencing technology and performed de novo assembly of 47,221 unigenes, with an average length of 655 bp. Our dataset provides the first repertoire of expressed sequences that could be used to identify and characterize transcripts potentially contributing to the adaptation of *F*. *danxiaensis* to extreme environments, in addition to providing data for further genetic studies. Based on this transcriptome, a *Firmiana*-specific whole-genome duplication event that occurred approximately 20 Mya was also identified, which may have played key roles in the speciation of *Firmiana* species.

## Materials and Methods

### RNA isolation and sequencing

Seedlings of *F*. *danxiaensis* were collected from Mount Danxia with the permission of the administrative committee of Mount Danxia National Park in April, 2009 and planted in the greenhouse of Sun Yat-sen University. After one year, fresh leaves were collected, and total RNA was isolated via the modified CTAB method [19]. Using Oligotex^TM^-dT30 (TaKaRa, Dalian, China), mRNA was extracted from the total RNA, then ultrasonically fragmented and converted to double-stranded cDNAs. After adding an “A” nucleotide at the 3′-end of the cDNAs, adapters were ligated to both ends, and the QIAquick Gel Extraction Kit (Qiagen, Hilden, German) was used to purify and collect cDNAs of approximately 215 bp in length. Finally, each amplified molecule was sequenced using Illumina sequencing technology to obtain short reads of 90 bp from both ends.

### Data filtering and de novo assembly

The raw reads were cleaned by removing reads containing unknown “N” bases or more than 10% bases with a Q value < 20 using custom Perl scripts. The cleaned reads were then de novo assembled into transcripts and unigenes using Trinity (r20140413) with the default parameters [20].

Setting an E-value cut-off of 1e^-5^ and up to 20 best hits, the assembled unigenes were subjected to BLASTX searches against the NCBI non-redundant (NR) protein database, and the protein sequences of *Arabidopsis thaliana* and two other malvaceous species (*Theobroma cacao* and *Gossypium raimondii*) downloaded from phytozome v 10.2 [21–24]. The Ortholog Hit Ratio (OHR) method developed by O’Neil et al. was used to assess the completeness of the assembled unigenes [25]. The OHR of each unigene was defined as the ratio of the number of bases in the BLASTX hit region to three times the length of the best-matched protein. Based on these values, the OHR for each unigene was computed with custom Perl scripts. An OHR ≥ indicates that the de novo-assembled unigene covers the entire matched protein, while an OHR < 1 indicates that the unigene is shorter than the matched protein.

### Functional annotation

To perform Gene Ontology (GO) annotation, the assembled unigenes were subjected to searches against the NR database using BLASTX. The results were then imported into Blast2GO to annotate the unigenes with Gene Ontology (GO) terms [26]. The annotations for each unigene were exported from Blast2GO and uploaded to the Web Gene Ontology Annotation Plot web tool (WEGO, http://wego.genomics.org.cn/cgi-bin/wego/index.pl) to run the GO functional classification.

Based on the best hit against the NR database, the coding region was extracted using custom Perl scripts and translated into the corresponding protein sequence with Transalign 1.0 [27]. After removing the sequences containing stop codons and those shorter than 50 amino acids with custom Perl scripts, these protein sequences were uploaded to webMGA http://weizhong-lab.ucsd.edu/metagenomic-analysis/server/), and aligned to the EuKaryotic Orthologous Groups (KOG) and Kyoto Encyclopedia of Genes and Genomes (KEGG) pathway databases (Setting an E-value cut-off of 1e^-5^) to predict and classify KOG and KEGG functions [28,29]. Based on the same procedures, functional annotation was performed for coding and protein sequences of *T*.*cacao* (downloaded from phytozome), and comparisons of the annotations between *F*. *danxiaensis* and *T*. *cacao* were performed.

### Detection of ancient whole-genome duplication events

For the above-mentioned coding sequences, all-by-all BLASTN searches were performed to identify duplicate pairs, with settings of a cut-off e-value of 1e^-5^ and a 40% sequence identity over at least 150 base pairs. Phylogenies for each gene family were constructed via single linkage clustering to reduce the multiplicative effects of multicopy gene families on *K*_*s*_ values [30]. Node *K*_*s*_ values were calculated using the YN00 method implemented in PAML [31,32]. Paralogous pairs showing *K*_*s*_ = 0 were removed from further analyses to reduce the possibility of identical genes or genes with alternative splicing. The *K*_*s*_ frequency in each interval with a size of 0.05 within the range [0, 2.0] was plotted. A mixture model of normal distributions was fit to the *K*_*s*_ distribution data via maximum likelihood using the EMMIX package [33]. For mixture model analyses, 1–10 normal distributions were fitted to the data with 100 random starts and 10 k-mean starts, and the Bayesian information criterion (BIC) and Akaike Information Criterion (AIC) were used to identify the number of normal distributions and select the best model fit to the data.

The relative age distribution of the duplicated genes was inferred from the observed distribution of synonymous distances. Coalescence estimates were obtained using an average synonymous substitution rate of 6.1 × 10^−9^ substitutions per synonymous site per year for angiosperms to date the paleopolyploidy event [34].

## Results and Discussion

### De novo assembly

**Fig 1:**
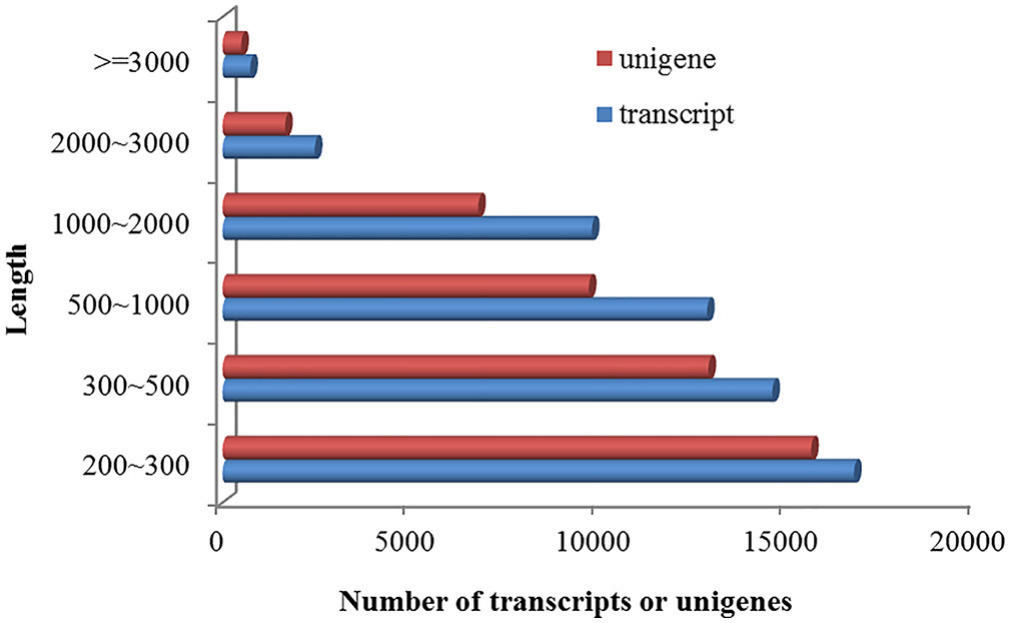
The length distribution of the assembled transcripts or unigenes.

A total of 11,977,779 × 2 (90 base) high-quality reads were obtained after data filtering and de novo assembled into 57,235 transcripts. The transcript length ranged from 200 bases to more than 3,000 bases, showing a total length of approximately 41 M, a mean length of 719 bp, and an N50 value of 1,105 bp (Fig 1). These transcripts were marginally AT rich (AT content: 58.1%) and represented 47,221 unigenes (average length: 655 bp; N50 value: 987 bp). The sum of the length of these unigenes was approximately 21 M, with a depth of coverage of 69.7 × (calculated by dividing the number of nucleotides in the cleaned reads by those in the unigenes). The raw data and the assembled transcripts were deposited as an NCBI BioProject (http://www.ncbi.nlm.nih.gov/bioproject/274165).

### Similarity analysis

**Fig 2:**
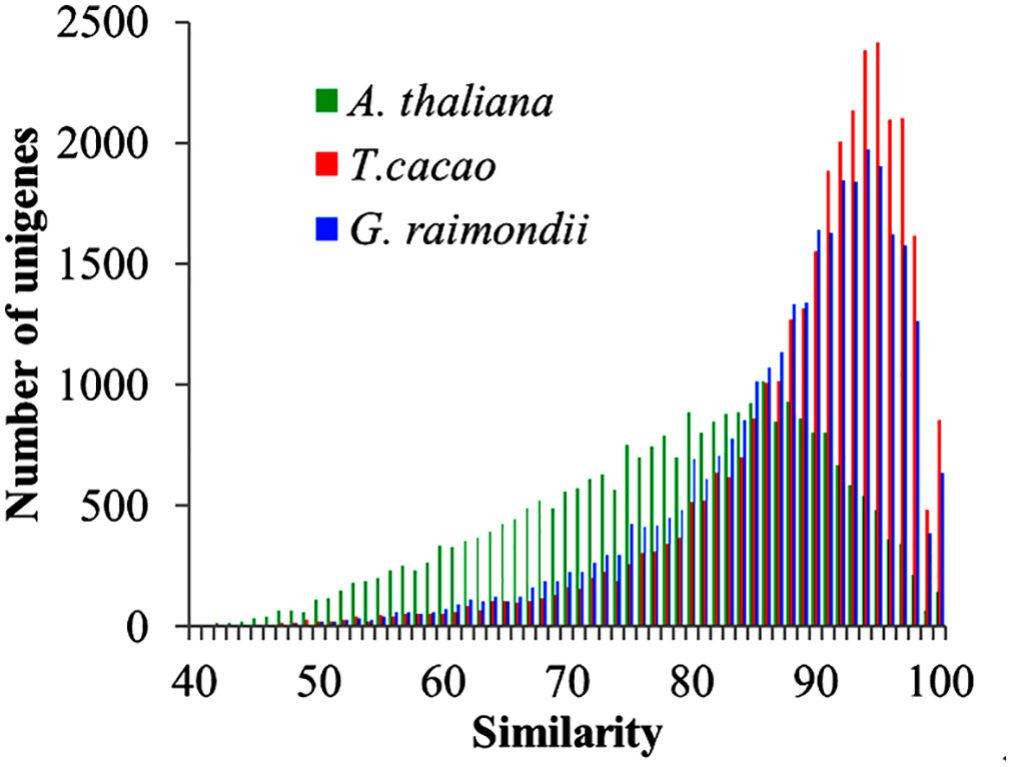
Similarity and number of matches to *A*. *thaliana*, *T*. *cacao* and *G*. *raimondii*.

**Fig 3:**
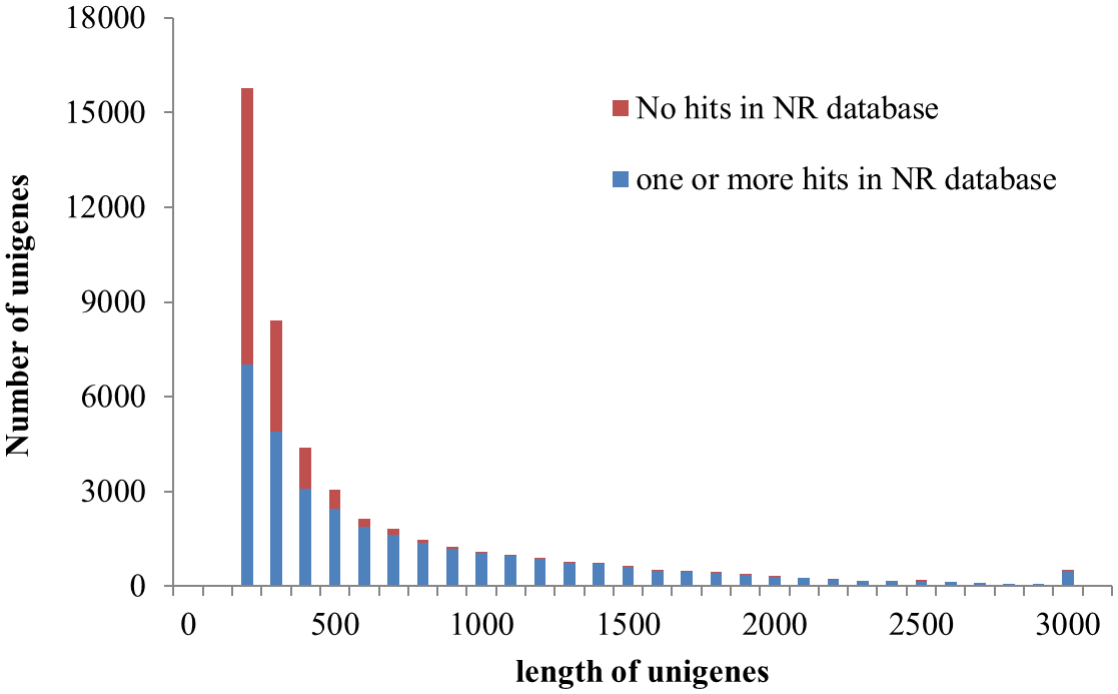
Length distribution of those unigenes unmatched to NR database.

A total of 47,211 unigenes were subjected to BLASTX similarity analysis, and 32,318 (68.5%) showed one or more hits in the NR database. For the rest (31.5%), more than 80% of them are shorter than 400bp in length (Fig 2), indicating that many of them may be too short to get hits, or lack a characterized protein domain, and some others may represent novel genes with uncharacterized functions [35–37]. Based on their top BLAST hits (highest score, S1 Table), a total of 24,370 unigenes were matched to hits in *Theobroma cacao*, 4,261 in *Gossypium arboretum*, and 460 in *Vitis vinifera*. All of the 30 species that provided the greatest numbers of top BLAST hits were plants, indicating extremely low contamination in our experiments. Further, a total of 32,275 unigenes showed significant matches to the protein databases of *T*. *cacao*, *G*. *raimondii* and *A*. *thaliana* (31,857 found in *T*. *cacao*, 31,081 in *G*. *arboretum*, 26,871 in *A*. *thaliana*, and 26,561 shared in these three species, Fig 3 and S1 Table). These results were not surprising, as *F*. *danxiaensis*, *T*. *cacao* and *G*. *arboretum* are all members of Malvaceae. These findings greatly contribute to the annotations of the transcriptome of *F*. *danxiaensis*.

**Fig 4:**
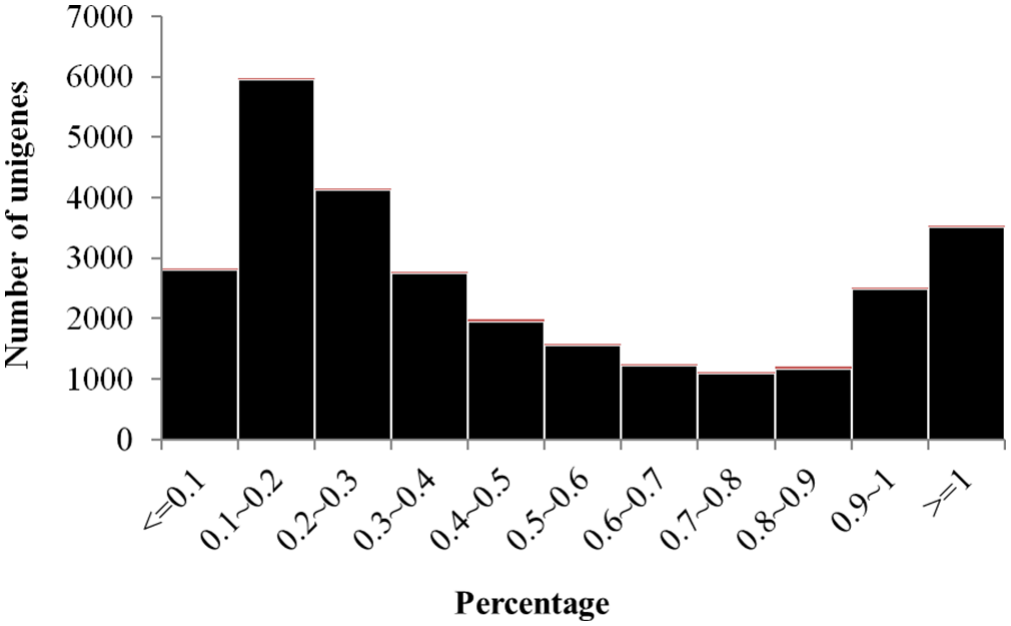
Length coverage distribution of *F*. *danxiaensis*’s transcriptome to known proteins of the NR database.

Based on their top blast hits, coding regions were obtained for 28,615 unigenes with a minimum length of 150 bases. Among them, 3,509 (12.3%) unigenes cover the complete length of their hits and can be considered to represent full-length transcripts, while 7,723 (27.0%) cover more than 75% of their hits, and 11,026 (38.5%) cover more than half of their hits (Fig 4). Overall, our dataset provided a large number of unigenes with high identity and coverage.

### Functional annotation

#### GO annotation

Based on the annotations against the NR database, a total of 18,288 unigenes were assigned to at least one of the three main GO categories (S1 Table): biological process (33,591 GO terms to 13,576 unigenes), molecular function (27,822 GO terms to 15,246 unigenes), and cellular component (15,506 GO terms to 9,889 unigenes).

The plant hormone abscisic acid (ABA) is the major player in mediating the adaptation of plants to stress [38]. In the transcriptome of *F*. *danxiaensis*, we obtained 125 unigenes assigned to the GO term “response to abscisic acid stimulus”. Furthermore, there are 214 unigenes assigned to “response to salt stress”, 141 to “response to cold”, 115 to “response to water deprivation”, and 143 to “response to oxidative stress”. In addition, we identified 7 unigenes assigned to “cellular response to nitrogen starvation”, 31 to “cellular response to phosphate starvation”, and 4 to “cellular response to potassium ion starvation”. The numbers of unigenes assigned to these GO terms in the transcriptome of *F*. *danxiaensis* were comparable to those in the genome of *T*. *cacao*, indicating the completeness of the assembled transcriptome. Further investigations based on these unigenes may help us to understand how *F*. *danxiaensis* survives in the barren soil on the rocky cliffs with low nitrogen, phosphate and potassium levels associated with the Danxia landform.

Cd pollution is known to cause severe public health problems. On Mount Danxia, the average concentration of Cd is 0.50 ± 0.16 (Chen *et al*. unpublished data), which is much higher than the average level (according to Heinrichs et al., it is 0.098 mg/kg in the lithosphere [39]). In the highly acidic soils of the Danxia landform, Cd could be more available under ionic and solvable conditions (0.35 ± 0.15 mg/kg, Chen *et al*. unpublished data), which may be toxic to or highly accumulated in local plant species [40]. In this transcriptome, we obtained 263 unigenes assigned to the “response to cadmium ion” term, which may help *F*. *danxiaensis* tolerant the high concentration of cadmium in the environment. Coincidently, some studies have shown that the leaves of *F*. *simplex* are high accumulators of Cd [41]. These unigenes obtained in the present study may provide keys to understand the molecular mechanism underlying this ability to accumulate Cd and further contribute to alleviating Cd pollution in natural environments.

**Fig 5:**
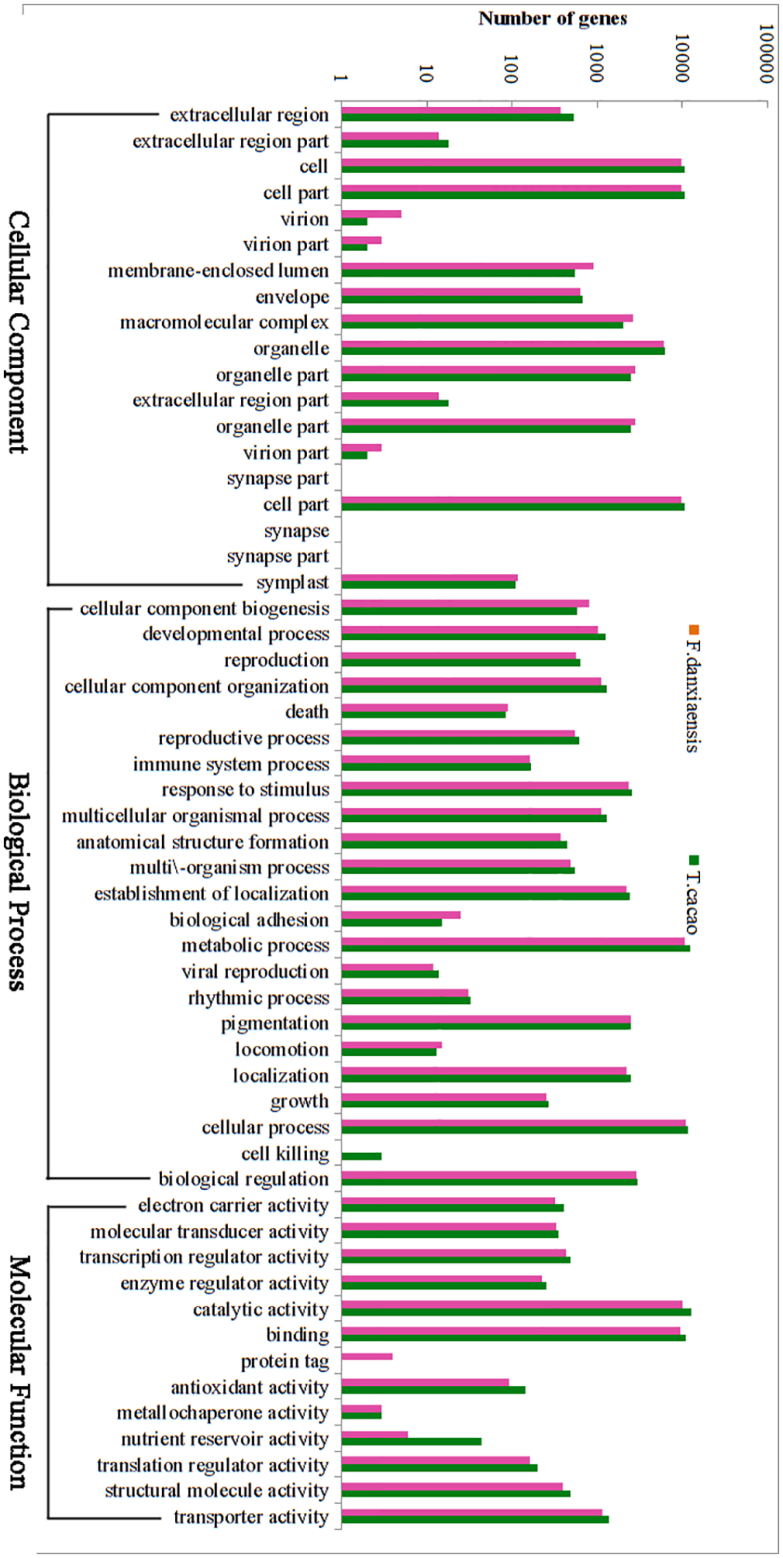
GO classification of the assembled unigenes of *F*. *danxiaensis* and the coding sequences of *T*. *cacao*.

According to WEGO, all of the GO terms were subdivided into 50 categories. Regarding the cellular component ontology, proteins related to the “cell,” “cell part,” and “organelle” categories were the best represented. Under the molecular function ontology, proteins assigned to the “binding” and “catalytic” terms were highly encoded. Among the various biological processes, the “metabolic process,” “response to stimulus” and “biological regulation” categories were found to show the greatest number of unigenes (more than 20% in the three main categories, Fig 5). A comparison between *F*. *danxiaensis* and *T*. *cacao* showed marginally more GO-annotated unigenes in *T*. *cacao* (22,619 GO-annotated proteins in total), yet the number of annotated unigenes in each GO category was close to each other and highly significantly correlated (r = 0.996, P < 0.001, Fig 5).

#### KOG annotation

**Fig 6:**
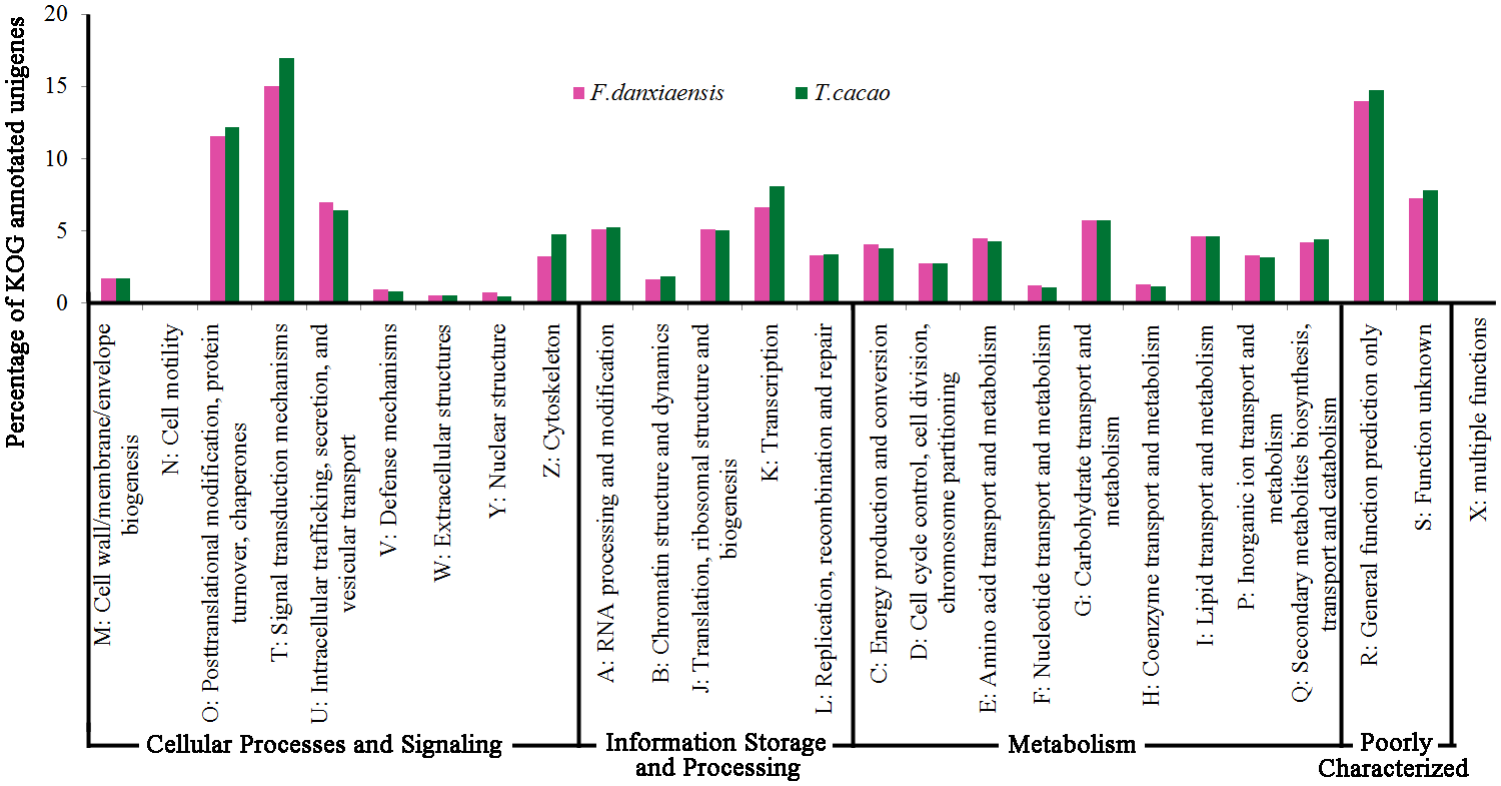
KOG functional classification of the assembled unigenes of *F*. *danxiaensis* and the protein sequences of *T*. *cacao*.

The coding regions obtained for the 28,615 unigenes were translated into proteins, among which 13,664 showed significant matches with the KOG databases and were classified into 26 KOG clusters (Fig 6, S1 Table). The top four categories with the greatest number of unigenes were “Signal transduction mechanisms” (2049), “General function prediction only” (1907), “Posttranslational modification, protein turnover, chaperones” (1581), and “Function unknown” (994). The four categories with the fewest unigenes were “Multiple function” (1), “Cell motility” (7), “Extracellular structures” (74) and “Nuclear structure” (99). Also, we identified 578 unigenes annotated to “Secondary metabolites biosynthesis, transport and catabolism”, which may play important roles in plant defense mechanisms [42].

Compared with KOG-annotations of *T*. *cacao* (A total of 44,404 proteins were used for annotation, and 24,067 obtained significant hits), the number of KOG-annotated unigenes in *F*. *danxiaensis* was obviously less (13,664), probably due to much less proteins used for analysis (28,615). However, the percentage of unigenes in each KOG cluster was rather similar and significantly correlated between these two species (r = 0.989, P < 0.001, Fig 6). This correlation was also found between *F*. *danxiaensis* and *Costus pictus* (r = 0.954, P < 0.001), and between *F*. *danxiaensis* and *Curcuma longa* (r = 0.930, P < 0.001), indicating that the numbers of genes in each KOG cluster may also be proportional to one another in different plant species [43, 44].

#### KEGG pathway annotation

**Fig 7:**
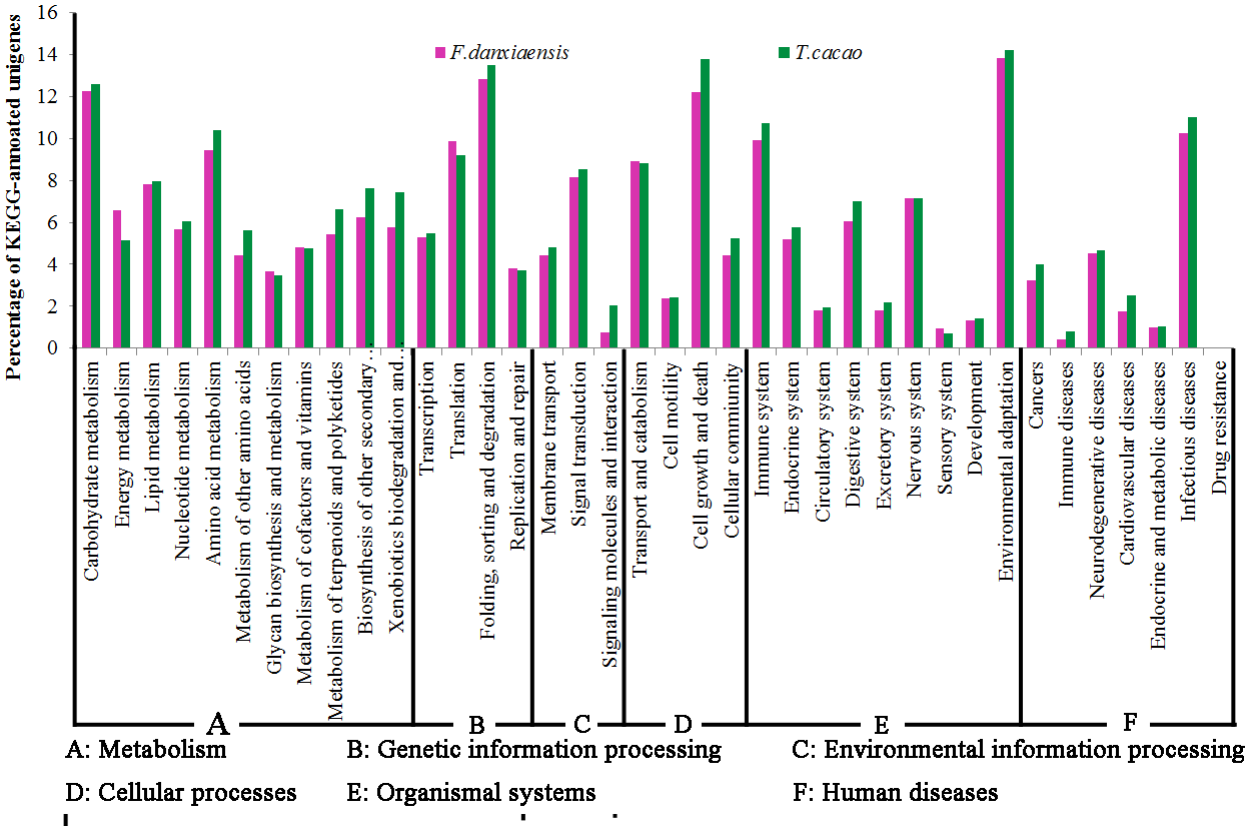
KEGG pathway assignment of the assembled unigenes of *F*. *danxiaensis* and the protein sequences of *T*. *cacao*.

A total of 13,692 unigenes were mapped to 288 KEGG pathways corresponding to six KEGG modules (Fig 7, S1 Table). Most of them involved in “Environmental adaptation” (1315 unigenes), “Folding, sorting and degradation” (1220), “Carbohydrate metabolism” (1165), and “Cell growth and death” (1162). Among the 1315 unigenes participating in “Environmental adaptation”, 1233 play roles in “plant-pathogen interaction” and 156 in “Circadian rhythm—plant”. Also, we identify many unigenes mapped to important pathways for the biosynthesis of secondary metabolites, such as 293 unigenes in “Phenylpropanoid biosynthesis”, 168 in “Phenylalanine metabolism” and 180 in “Flavonoid biosynthesis” [45,46]. These unigenes may greatly contribute to the survivor and success of *Firmiana* species in extreme environments. By comparison with *T*. *cacao* (26,632 KEGG-annotated proteins), the number of annotated unigenes in most KEGG pathways was also less in *F*. *danxiaensis*, yet both species possessed similar percentages of annotated genes in the vast majority of KEGG pathways (Fig 7). Altogether, these nearly identical patterns observed in the GO, KOG, and KEGG annotations between *F*. *danxiaensis* and *T*. *cacao* suggested the completeness of the assembled transcriptome of *F*. *danxiaensis*.

### Paleopolyploid events occurring in *Firmiana*

**Fig 8:**
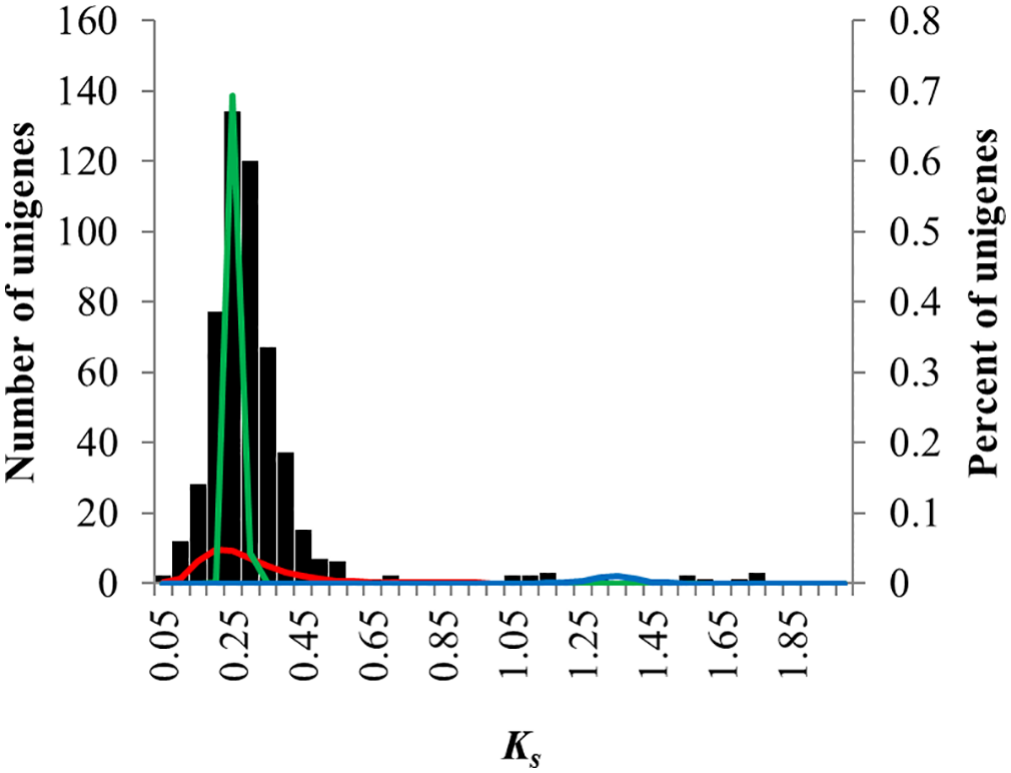
*K*_*s*_ frequency plots for paralogous gene pairs from *F*. *danxiaensis*. *K*_*s*_ distribution components estimated using EMMIX are superimposed on the histogram. These components are hypothesized to represent background gene duplications (green) and gene duplication associated with polyploidy events (red).

**Fig 9:**
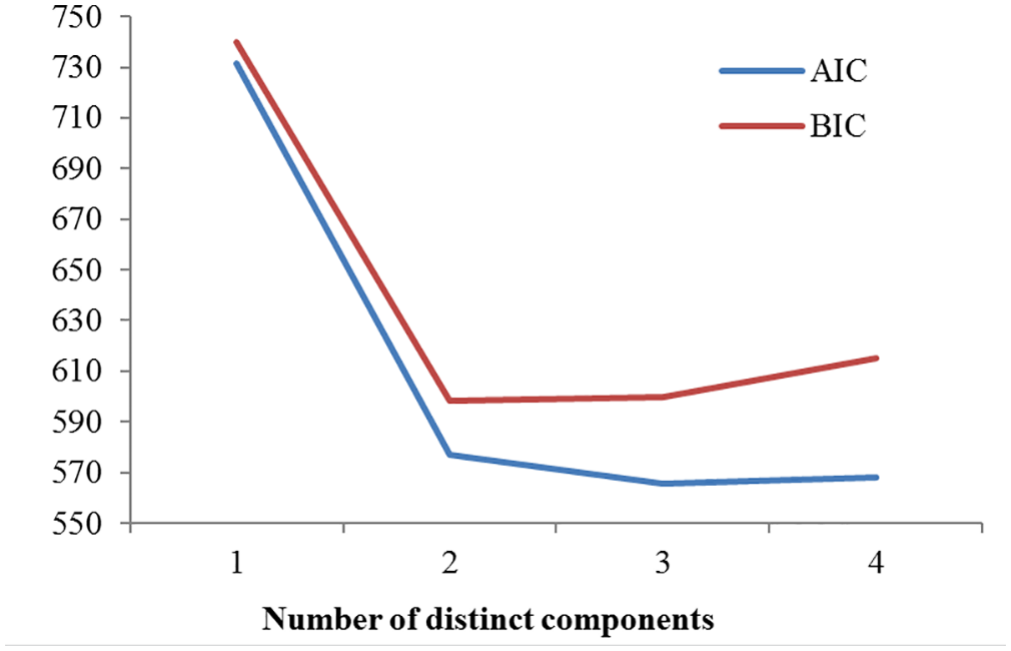
Estimated AIC and BIC values under various number of distinct components with EMMIX program.

A total of 529 paralogous genes were identified (*K*_*s*_ < 2). The average *K*_*s*_ and *K*_*a*_ values between these paralogous genes were 0.322 ± 0.262 and 0.070 ± 0.038, respectively. The *K*_*s*_ plot for paralogous genes showed one obvious peak, where *K*_*s*_ = 0.25 (Fig 8). Mixture model analyses revealed that two distinct components were determined for the lowest BIC (Fig 9, Table 1): the first component (centered at approximately 0.25) was considered to be indicative of a single duplication event occurring approximately 20 Mya, and the second component (centered at approximately 0.29) exhibited a large standard deviation (0.828) and was considered to reflect numerous independent duplications across time (background gene duplication) (Table 1). Three distinct components were revealed when the AIC was lowest and the BIC was slightly greater than its lowest value (Fig 9, Table 1): the first component (centered at approximately 0.21) was deemed to represent a background gene duplication, as it also showed a large standard deviation, and the second component (centered at approximately 0.25) agreed with the first component when the BIC was lowest, while the third component (centered approximately 1.33) indicated another duplication event occurring approximately 109 Mya (Table 1).

**Table 1:** Distribution and divergence estimates.

No. of distributions	Natural log (Ln) of the mean ^a^	Median of distribution ^b^	Divergnce estimate (Mya)
2	-1.383 (0.074)	0.251	20.6
	-1.2264 (0.828)	0.293	
3	-1.518 (0.402)	0.219	
	-1.366 (0.068)	0.255	20.9
	0.287 (0.044)	1.332	109.0

^a^ The median was determined from back-transformation of the natural log (Ln) of the mean (standard deviation in parentheses)

^b^ Divergence estimates were calculated from the median of the distribution (Mya, million years ago)

Thus, our study revealed two whole-genome duplication events that have occurred in *F*. *danxiaensis*: the ancient duplication event might have occurred approximately 109 Mya, while the younger event was predicted to have occurred 20 Mya. The ancient duplication event supported previous studies indicating that a whole-genome duplication event occurred approximately 117 Mya throughout core eudicots [47], which also suggested that our data are reliable. The young duplication event occurring approximately 20 Mya could be restricted to *Firmiana* species, as in fossil records, the leaves of *Firmiana* species have been identified from the Lower Oligocene, while the leaves and fruits have been documented from the Middle Miocene [48]. Furthermore, this young duplication event co-occurred with the rise of the Qinghai-Tibetan Plateau, which could have driven the divergence of many plant species through polyploidy [49–51]. Considering the distribution of *Firmiana* species, it is possible that the rise of the Qinghai-Tibetan Plateau may have also contributed to the polyploidy of basal *Firmiana* species and, subsequently, to the widespread distribution of *F*. *simplex* and *F*. *colorata* as well as the speciation of other endemic *Firmiana* species adapted to local environments.

## Supporting Information

S1 Table Annotation information for the transcriptome of *F*. *danxiaensis*. (XLS)
